# Fatiguing Trunk Flexor Exercise Decreases Pain Sensitivity in Postpartum Women

**DOI:** 10.3389/fphys.2019.00315

**Published:** 2019-03-26

**Authors:** Rita Deering, Tatyana Pashibin, Meredith Cruz, Sandra K. Hunter, Marie Hoeger Bement

**Affiliations:** ^1^Department of Physical Therapy, Marquette University, Milwaukee, WI, United States; ^2^William S. Middleton Veterans Hospital, Madison, WI, United States; ^3^Department of Orthopedics and Rehabilitation, School of Medicine and Public Health, University of Wisconsin-Madison, Madison, WI, United States; ^4^Department of Obstetrics and Gynecology, Medical College of Wisconsin, Milwaukee, WI, United States

**Keywords:** exercise-induced hypoalgesia, pregnancy, pressure pain thresholds, muscle thickness, sex differences

## Abstract

**Background:**

Low back pain (LBP) is common in the general population and among postpartum women. Abdominal muscle exercise is often used to treat LBP, but it is unknown if fatiguing abdominal muscle exercise can produce exercise-induced hypoalgesia (EIH).

**Objectives:**

To assess pressure pain thresholds (PPTs) at rest and following fatiguing trunk flexor exercise (EIH) in (1) nulligravid and postpartum women to evaluate the impact of pregnancy and childbirth and (2) nulligravid women and men to examine sex differences.

**Methods:**

Seventy healthy adults (31 postpartum women, 23 nulligravid women, 16 men) participated. Postpartum and nulligravid women were tested twice (16–18 weeks apart) to identify changes in EIH with postpartum recovery. PPTs were measured at the nailbed and superior rectus abdominis before and after exercise to investigate systemic and local EIH, respectively. Rectus abdominis muscle thickness was assessed with ultrasound.

**Results:**

Postpartum women reported lower PPTs than nulligravid women at the abdomen (*p* < 0.05) whereas postpartum women had lower PPTs at the nailbed during the first session only. Men reported higher nailbed PPTs (*p* = 0.047) and similar PPTs at the abdomen than women (*p* = 0.294). All groups demonstrated EIH at the abdomen (*p* < 0.05). Systemic EIH was absent in postpartum and nulligravid women (*p* > 0.05), while men demonstrated hyperalgesia. Local EIH was positively associated with muscle thickness for men and women, which was not significant at the second timepoint.

**Limitations:**

Acute exercise response may not reflect changes that occur with exercise training.

**Conclusion:**

Fatiguing trunk flexor exercise produced local EIH for all groups including postpartum and nulligravid women. Clinically, trunk exercises may be useful for acute pain relief for clinical populations that are characterized by pain and/or weakness in the abdominal region muscles in populations with abdominal pain syndromes.

## Introduction

Approximately 30% of adults in the United States experience chronic pain, with the low back being the most commonly reported location (Johannes et al., 2010). Low back pain (LBP) has also been identified as the leading cause of years lived with disability globally (Vos et al., 2012), and women are more likely than men to experience chronic LBP that impacts activities of daily living (Chenot et al., 2008). In addition, LBP frequently occurs with pregnancy, with approximately 75% of pregnant women reporting low back and/or pelvic girdle pain (Wang et al., 2004; Wu et al., 2004). Roughly one out of four women with LBP during pregnancy will continue to have pain after childbirth (Albert et al., 2000; Robinson et al., 2010), and up to 20% of these women have pain that interferes with performance of activities of daily living 3 years after childbirth (Albert et al., 2001; Norén et al., 2002). Furthermore, prolonged pain can lead to central sensitization, which is an increased responsiveness of nociceptive neurons in the central nervous system (International Association for the Study of Pain), making early management of pain an important factor in the prevention of chronic pain syndromes and disability.

Several studies have identified impaired function of the abdominal muscles in individuals with back pain. Smidt et al. (1983) found that individuals with LBP demonstrated lower isometric and isokinetic trunk flexion torques and were more susceptible to muscle fatigue of the abdominals compared with individuals without LBP. Postpartum women have been shown to have lower isometric trunk flexion strength and increased fatigability of both the trunk flexor muscles and lumbopelvic stabilizing muscles up to 26 weeks postpartum as compared to women who have never been pregnant (Deering et al., 2018a,b), which may contribute to LBP in this population. In postpartum women, increased inter-recti distance, suggesting compromised fascial integrity, has also been associated with low back and pelvic pain (Parker and Millar, 2008). These findings suggest that dysfunction of the abdominal muscles may play a role in the etiology of LBP, with rehabilitation implications including the response to exercise.

Pain can also lead to fear avoidance behaviors leading to further reductions in physical activity levels and muscle weakness; perpetuating a cycle of weakness, instability, pain, and reduced movement (Gutke et al., 2011). In contrast, exercise has been shown to be a non-pharmacologic intervention to decrease pain sensitivity in healthy individuals and patient populations (Naugle et al., 2012). The reduction in pain perception following exercise is known as exercise-induced hypoalgesia (EIH). Pressure pain thresholds (PPTs), which are the minimum intensity of a stimulus that is perceived as painful, are frequently used to assess EIH; an increase in PPTs following exercise is indicative of EIH (Hoeger Bement et al., 2008; Naugle et al., 2012).

EIH can occur locally at the exercising muscle as well as systemically at more distal sites such as the nail bed. The magnitude of EIH is dependent upon both the intensity and duration of exercise (Hoeger Bement et al., 2008; Naugle et al., 2012); greater pain relief occurs with fatiguing contractions (Hoeger Bement et al., 2008). Emerging evidence also shows that women may experience greater EIH than men, although this may be due to differences in baseline (pre-exercise) pain (Koltyn et al., 2001; Lemley et al., 2016).

Because postpartum women are at high risk of LBP and have low strength and endurance of the abdominal muscles compared with nulligravid women (Deering et al., 2018a,b), and as abdominal muscle exercise is typically used in the treatment of LBP for both men and women (Richardson et al., 1999; Hodges, 2003; Bastiaenen et al., 2004; Koumantakis et al., 2005; Pennick, 2007; Vleeming et al., 2008; Liddle and Pennick, 2015), we evaluated PPTs before and after fatiguing exercise of the trunk flexor muscles in (1) postpartum and nulligravid women (controls) to examine the impact of pregnancy and childbirth on pain thresholds and EIH, and (2) healthy men and nulligravid women to examine potential sex differences in pain thresholds and EIH (Koltyn et al., 2001) following abdominal muscle exercise. Postpartum and nulligravid women were tested twice, during the initial (8–10 weeks) postpartum period and follow-up (24–26 weeks) postpartum period, to determine if pain perception and response to exercise changes with the length of the postpartum period. The initial testing time point (8–10 weeks postpartum) was chosen due to many women in the United States receiving only 6–8 weeks of maternity leave (Vahratian and Johnson, 2009). Women who delivered vaginally or via Cesarean delivery were included, as both delivery methods are associated with some degree of pain and inflammation, whether from perineal/pelvic floor muscle injury or pelvic joint trauma from vaginal birth or surgical pain from Cesarean delivery, which can activate nociceptors (Chimenti et al., 2018). We hypothesized that (1) postpartum women would report lower resting PPTs than nulligravid women, and that both groups would demonstrate an increase in PPTs following fatiguing exercise (EIH) and (2) women would report lower PPTs at rest than men, and that both men and women would demonstrate EIH.

## Materials and Methods

Seventy healthy adults participated in the study. To examine the impact of pregnancy and childbirth on pain perception and EIH in response to abdominal muscle exercise (protocol 1), 31 postpartum women and 22 nulligravid women participated. To examine potential sex differences (protocol 2), 16 men and 19 women participated. Eighteen women participated in both protocols. During protocol 1, postpartum women were tested twice at the initial postpartum period (between 8 and 10 weeks) and follow-up at 24–26 weeks postpartum. Nulligravid women were tested, similarly, at two timepoints, separated by 16–18 weeks to match the testing time points of the postpartum women.

For protocol 1, 27 women between 8 and 10 weeks postpartum (31.2 ± 5.2 years; Vaginal delivery *n* = 17, Cesarean delivery *n* = 10) and 14 nulligravid women (25.8 ± 5.3 years) completed the initial time point of testing. At the follow up time point (24–26 weeks postpartum), 26 postpartum women (31.4 ± 4.8 years; Vaginal delivery *n* = 15, Cesarean delivery *n* = 11) and 14 nulligravid women (25.8 ± 6.1 years) completed the protocol. Twenty-two postpartum women (vaginal delivery *n* = 14, Cesarean delivery *n* = 8) and six nulligravid women completed both time points.

### Protocol Overview

All participants completed two experimental sessions at each time point of testing. Thus, participants in protocol 1 completed four experimental sessions (initial and follow-up postpartum period), whereas participants in protocol 2 completed two experimental sessions. The two protocols were identical except for minor deviations as outlined in the methods. During session one for both protocols, participants were familiarized to the pressure algometer (AlgoMed) at the nailbed of the left middle finger, completed ultrasound imaging of the abdominal muscles, and measurements of height and weight. Participants from protocol one also underwent assessment of PPTs at the lower abdomen (see PPT section for details).

During session two for both protocols, participants completed multiple questionnaires (Physical Activity Questionnaire, Oswestry Disability Index, McGill Short Form Pain Questionnaire, Pain Catastrophizing Survey, and the Fear Avoidance Beliefs Questionnaire) and performed the fatiguing trunk flexor exercise protocol. PPTs were measured before and after the exercise protocol at the left superior rectus abdominis muscle (to assess EIH local to the exercising muscle) and nailbed (to assess systemic EIH). The study was approved by the Institutional Review Boards at Marquette University and the Medical College of Wisconsin, and the Office of Clinical Research and Innovative Care Compliance at Froedtert Hospital. All participants provided written informed consent prior to study enrollment.

### Fatiguing Trunk Flexor Exercise

Participants performed an intermittent isometric fatiguing trunk flexion task while seated upright in the Biodex dynamometer (Biodex Medical, Shirley, NY, United States). Trunk flexion maximal voluntary contractions (MVC) were performed prior to initiating the exercise protocol to determine maximal strength. At least three MVCs were performed, with a minimum of 1 min of rest between contractions, until two contractions were within 5% of each other to ensure true MVCs were obtained. The highest MVC was used to calculate the exercise intensity. The exercise protocol involved performance of trunk flexion contractions at 50% of MVC for 6 s with 4 s rest between contractions and 1 MVC every minute and at task failure (Deering et al., 2017). Task failure was defined as submaximal torque less than the 50% MVC target line for 3 s of the 6 s contraction or MVC strength less than or equal to 50% of baseline MVC.

### Pressure Pain Thresholds (PPTs)

PPTs were assessed using a computerized pressure algometer with a 1 cm^2^ rubber tip (Medoc Ltd., Yishai, Israel). PPTs were performed, before and within 5 min after the trunk flexor fatiguing exercise protocol while reclined in the Biodex dynamometer, at the nailbed of the left middle finger and at the left upper rectus abdominis (5 cm above and 2 cm lateral to the umbilicus). Three trials were performed at each site with an inter-stimulus interval of 10 s at a rate of 10 kPa/s. Participants were instructed to press a timing device “as soon as pressure changes to pain.” While testing the abdominal muscle site, participants received the same instructions with the added instruction to breathe normally and not to press their abdomen out against the algometer. Pain thresholds were recorded for all three trials and averaged. Change in PPT was quantified in absolute (post-exercise PPT minus pre-exercise PPT) and relative [(post-exercise PPT minus pre-exercise PPT)/pre-exercise PPT] values.

For protocol 1, PPTs were also performed at the lower abdomen (during first session). Postpartum women who underwent Cesarean delivery were tested at the midpoint of their surgical scar, and all women in this group did have a Pfannenstiel (transverse) incision. Postpartum women who experienced a vaginal delivery and nulligravid women were tested in the midline of the abdomen, where a Pfannenstiel incision would be performed (approximately two finger widths above the pubic symphysis) (Mathai and Hofmeyr, 2007). Three PPT trials were performed and averaged.

### Ultrasound Imaging of the Abdominal Muscles

Muscle thickness measurements of the right rectus abdominis were recorded at 2.5 cm above the umbilicus with a GE vivid e ultrasound machine (GE Healthcare, Little Chalfont, United Kingdom; 8LRS transducer). The full width of the rectus abdominis was scanned and the measurement was taken in the region that visually appeared to be the thickest at end expiration (Teyhen et al., 2007; Deering et al., 2017, 2018a,b).

### Questionnaires: Pain and Fear Avoidance

Pain assessments included McGill Short Form Pain Questionnaire (Melzack, 1987), Pain Catastrophizing Scale (Osman et al., 1997, 2000), and the Fear Avoidance Beliefs Questionnaire (Waddell et al., 1993). LBP related disability was assessed with the Oswestry Disability Index (Fairbank et al., 1980; Fairbank and Pynsent, 2000; Fairbank, 2014).

### Physical Activity

Physical activity at the time of testing was quantified with triaxial accelerometers (ActiGraph) worn around the waist for 4 days, inclusive of 2 weekend days. Average minutes of moderate intensity physical activity per day was calculated with ActiLife software.

Participants also completed a Physical Activity Questionnaire to estimate physical activity, represented as metabolic equivalents per hour per week, over the previous 12 months (Kriska et al., 1990; Deering et al., 2017).

### Statistical Analysis

Power analysis was conducted using G Power software, which indicated the need for 18 subjects per group to achieve 95% power with alpha level of 0.05. Independent *t*-tests compared subject characteristics and baseline PPTs between groups (postpartum and nulligravid) and sexes. Questionnaires with ordinal scales were compared between groups and sexes using the Mann–Whitney *U*-test. Change in PPTs following the exercise protocol were analyzed with repeated measures analysis of variance (ANOVA) over time (pre-post exercise) with group (postpartum vs. nulligravid) or sex as a between-subject factor. Correlation analysis between change in PPTs (post-pre) at the rectus abdominis muscle and muscle thickness was conducted with Spearman’s rho non-parametric correlation due to non-normal distribution of ultrasound data. Pearson correlation was used to explore the relationship between baseline pain and pain response to exercise at both the nailbed and the abdomen. Significance was identified at *p* < 0.05. Data is presented in the text and tables as means ± standard deviation (SD).

## Results

### Protocol One: Postpartum and Nulligravid Women

Subject characteristics, including weight, body mass index (BMI), trunk flexor strength and fatigability, and physical activity levels are presented in Table 1.

**Table 1:** Subject characteristics: nulligravid and postpartum.

	Initial (8–10 weeks postpartum)		Follow up (24–26 weeks postpartum)	
	Nulligravid (*n* = 14)	Postpartum (*n* = 27)	Nulligravid (*n* = 14)	Postpartum (*n* = 26)
Age (years)	25.8 ± 5.3	31.2 ± 5.2^∗^	25.8 ± 6.1	31.4 ± 4.8^∗^
Height (cm)	166.9 ± 7.4	164.3 ± 4.6	166.1 ± 8.6	163.8 ± 4.8
Weight (kg)	63.8 ± 13.1	75.6 ± 12.8^∗^	63.3 ± 8.0	70.7 ± 13.4^∗^
BMI (kg/m^2^)	22.8 ± 4.0	28.1 ± 4.8^∗^	22.8 ± 2.2	26.7 ± 4.9^∗^
McGill pain intensity (cm)	0.5 ± 1.1	0.3 ± 0.7	0.3 ± 0.7	0.9 ± 2.0
Oswestry (%)	1.1 ± 2.6	4.3 ± 5.4^∗^	0.9 ± 1.9	5.0 ± 7.3
Fear Avoidance Beliefs Questionnaire (AU)	0.3 ± 0.6	7.7 ± 10.3^∗^	0.5 ± 1.3	7.7 ± 12.3
Pain Catastrophizing Scale (AU)	9.9 ± 6.7	9.8 ± 9.1	6.1 ± 6.1	9.0 ± 9.4
Rectus abdominis muscle thickness (cm)	1.0 ± 0.2	0.8 ± 0.2^∗^	1.0 ± 0.1	0.8 ± 0.1^∗^
Trunk flexor MVC (Nm)	47.4 ± 26.8	27.6 ± 11.5^∗^	44.5 ± 17.2	23.9 ± 10.2^∗^
Trunk flexor time to task failure (s)	655.7 ± 336.3	191.8 ± 161.1^∗^	623.6 ± 405.5	290.7 ± 169.7^∗^
Self-reported physical activity over the previous 12 months (MET^∗^hr^∗^week^-1^)	44.2 ± 29.0 (*n* = 13)	23.1 ± 19.7^∗^ (*n* = 25)	30.3 ± 21.6 (*n* = 14)	15.6 ± 17.9^∗^ (*n* = 23)
Average minutes/day of moderate physical activity (accelerometer)	47.7 ± 25.0 (*n* = 8)	19.3 ± 19.2^∗^ (*n* = 19)	29.3 ± 14.1 (*n* = 8)	16.4 ± 11.2^∗^ (*n* = 11)

*^∗^Indicates p < 0.05. cm, centimeters; kg, kilograms; m, meter; Nm, Newton meters; s, seconds; MET, Metabolic equivalents; hr, hour.*

#### Pfannenstiel Site PPTs (Experimental Session 1)

No difference was noted in Pfannenstiel site PPTs between women who had a vaginal delivery and women who had a Cesarean delivery at 8–10 weeks postpartum (127.4 ± 53.2 vs. 103.8 ± 44.7, respectively, *p* = 0.251) or 24–26 weeks postpartum (112.3 ± 52.7 vs. 110.3 ± 46.5, respectively, *p* = 0.922), so both groups were combined. At the initial time point (8–10 weeks postpartum), postpartum women were more sensitive to pain (i.e., lower PPTs) than nulligravid women at the pfannenstiel site (*p* < 0.001; Table 2). At the follow up timepoint (24–26 weeks postpartum), postpartum women continued to demonstrate heightened sensitivity to pain at the pfannenstiel site (*p* = 0.001; Table 2).

**Table 2:** Experimental pain perception.

	Initial (8–10 weeks postpartum)		Follow up (24–26 weeks postpartum)		Protocol 2 (sex differences)	
	Nulligravid (*n* = 14)	Postpartum (*n* = 27)	Nulligravid (*n* = 14)	Postpartum (*n* = 26)	Men (*n* = 16)	Women (*n* = 19)
Pfannenstiel site PPT (kPa)	191.5 ± 43.8	118.7 ± 50.7^*^	178.2 ± 51.5	111.5 ± 49.2^*^	N/A	N/A
Baseline nailbed PPT (kPa)	260.7 ± 69.6	188.4 ± 105.6^*^	226.1 ± 107.6	190.9 ± 106.3	317.8 ± 162.2	228.2 ± 90.1^*^
Baseline superior rectus abdominis PPT (kPa)	186.9 ± 84.4	125.7 ± 62.7^*^	171.9 ± 79.4	115.2 ± 54.5^*^	217.7 ± 110.4	181.5 ± 90.3
Absolute change in PPT at nailbed (kPa)	-2.6 ± 32.9	6.3 ± 42.3	-12.8 ± 31.5	-10.6 ± 37.4	-29.8 ± 46.4	4.5 ± 27.5^*^
Absolute change in PPT at superior rectus abdominis (kPa)	27.1 ± 47.0	13.4 ± 27.0	10.5 ± 35.3	16.7 ± 19.4	27.9 ± 39.8	19.7 ± 41.8
Relative change in PPT at nailbed (%)	-0.6 ± 13.0	2.6 ± 24.6	-5.6 ± 20.1	-1.8 ± 22.3	-7.9 ± 17.4	0.9 ± 18.7
Relative change in PPT at superior rectus abdominis (%)	18.7 ± 21.7	10.7 ± 19.4	7.3 ± 19.0	16.6 ± 19.9	16.4 ± 20.6	13.6 ± 20.6

*^∗^Indicates p < 0.05. PPT, Pressure Pain Threshold; kPa, kilopascals; N/A, Not Assessed.*

#### Baseline PPTs Prior to Fatiguing Trunk Flexor Exercise (Experimental Session 2)

There was no difference in baseline PPTs between women who had a vaginal delivery and women who had a Cesarean delivery at 8–10 weeks postpartum (NAILBED: 169.2 ± 87.5 vs. 221.1 ± 129.4, respectively, *p* = 0.225; SUPERIOR RECTUS ABDOMINIS: 117.2 ± 55.7 vs. 140.1 ± 74.1, respectively, *p* = 0.371) or 24–26 weeks postpartum (NAILBED: 157.9 ± 89.9 vs. 235.9 ± 114.3, respectively, *p* = 0.063; SUPERIOR RECTUS ABDOMINIS: 101.0 ± 41.2 vs. 134.5 ± 65.9, respectively, *p* = 0.124), so both groups were combined. At the initial timepoint, postpartum women had lower PPTs than nulligravid women at the nailbed (*p* = 0.026; Table 2) and the superior rectus abdominis site (*p* = 0.031; Table 2). By 26 weeks postpartum, there was no difference in baseline pain at the nailbed between postpartum and nulligravid women (*p* = 0.326; Table 2), but postpartum women continued to be more sensitive to pain at the superior rectus abdominis site (*p* = 0.027; Table 2) compared with the nulligravid women.

#### PPTs Before and After Fatiguing Trunk Flexor Exercise

**FIGURE 1:**
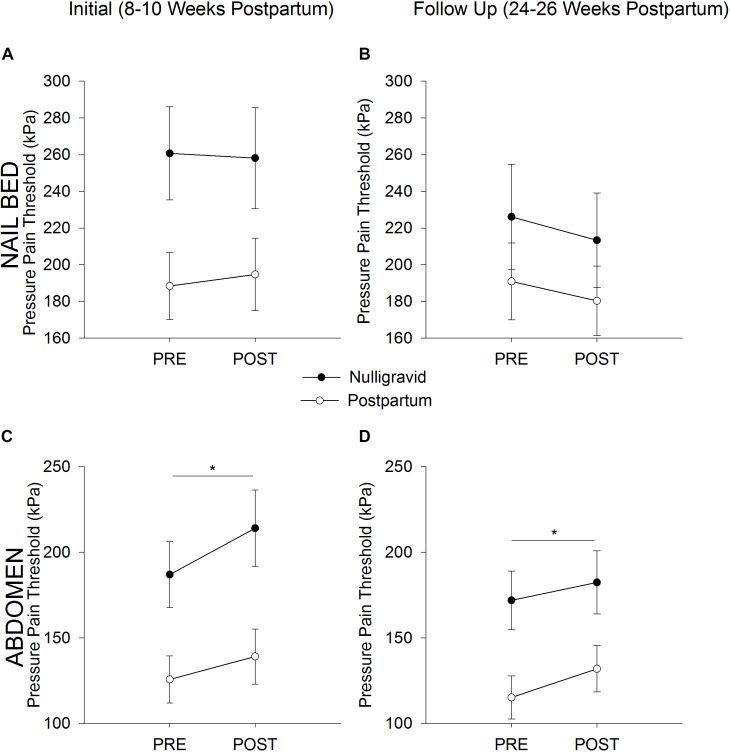
Postpartum vs. Nulligravid PPTs. Pressure Pain Threshold before (Pre) and after (Post) Exercise in Nulligravid and Postpartum Women at the Nailbed **(A,B)** and Abdomen **(C,D)**. Neither postpartum nor nulligravid women demonstrated a change in PPT at the nailbed following fatiguing trunk flexor exercise at the initial **(A)** or follow-up **(B)** time points. Postpartum women had lower PPTs at the nailbed than nulligravid women at 8–10 weeks postpartum **(A)** but had similar PPTs as nulligravid women 24–26 weeks after childbirth **(B)** due to a decline in nulligravid PPTs from initial to follow up. Both postpartum and nulligravid women demonstrated EIH at the superior rectus abdominis site following fatiguing trunk flexor exercise at both the initial **(C)** and follow up **(D)** time points. Postpartum women had lower PPTs than nulligravid women at the abdomen at 8–10 weeks postpartum **(C)** and 24–26 weeks postpartum **(D)**. ^∗^ indicates *p* < 0.05 (time effect).

At the initial timepoint, women who delivered vaginally and women who delivered via Cesarean section had similar pain responses to exercise at the nailbed (time *p* = 0.489; time × delivery type *p* = 0.917; delivery type *p* = 0.246) and the upper abdomen (time *p* = 0.019; time × delivery type *p* = 0.778; delivery type *p* = 0.382), so both delivery types were combined into one postpartum group. Nulligravid and postpartum women demonstrated an increase in PPT (i.e., local EIH) at the superior rectus abdominis following fatiguing trunk flexor exercise (time *p* = 0.001; time × group *p* = 0.241; Figure 1C). At the nailbed, postpartum women and nulligravid women demonstrated no change in PPT following exercise (time *p* = 0.780; time × group *p* = 0.498; Figure 1A). There were no differences in absolute or relative changes in PPTs after fatiguing exercise between postpartum and nulligravid women at the nailbed or superior rectus abdominis site at the initial or follow up timepoints (*p* > 0.05; Table 2).

At the second timepoint, no difference was noted in the pain response to exercise in women who delivered vaginally or via Cesarean section at the nailbed (time *p* = 0.106; time × delivery type *p* = 0.163; delivery type *p* = 0.089) or the upper abdomen (time *p* < 0.001; time × delivery type *p* = 0.650; delivery type *p* = 0.165), so both delivery types were combined into one postpartum group. Postpartum and nulligravid women had an increase in PPT at the superior rectus abdominis following fatiguing trunk flexor exercise (time *p* = 0.003; time × group *p* = 0.472; Figure 1D). At the nailbed, PPTs were unchanged following fatiguing trunk flexor exercise (time *p* = 0.054; time × group *p* = 0.854; Figure 1B).

#### Associations

At the initial timepoint, EIH (increase in PPTs following exercise) at the superior rectus abdominis site was positively correlated with thickness of the rectus abdominis muscle (*r* = 0.321, *p* = 0.026); women with thicker abdominal muscles experienced greater local EIH. At the second timepoint only, exercise-induced changes in PPT at the nailbed was associated with baseline PPT at the nailbed (*r* = -0.396, *p* = 0.025); women who demonstrated greater baseline PPT at the nailbed reported less EIH than women with lower baseline PPT. Baseline PPT at the superior rectus abdominis was not associated with EIH at the superior rectus abdominis (*r* = -0.056, *p* = 0.733).

### Protocol Two: Men and Women

Subject characteristics, including weight, BMI, trunk flexor strength and fatigability, and physical activity levels are presented in Table 3.

**Table 3:** Subject characteristics: men vs. nulligravid women.

	Men (*n* = 16)	Nulligravid women (*n* = 19)
Age (years)	24.1 ± 6.6	24.4 ± 4.9
Height (cm)	176.8 ± 7.4	167.1 ± 9.1^∗^
Weight (kg)	72.7 ± 8.5	65.6 ± 12.2
BMI (kg/m^2^)	23.0 ± 2.3	23.3 ± 3.7
McGill pain intensity (cm)	0.04 ± 0.2	0.4 ± 1.0
Oswestry (%)	2.1 ± 3.2	0.9 ± 2.0
Fear Avoidance Beliefs Questionnaire (AU)	1.9 ± 5.6	0.4 ± 0.8
Pain Catastrophizing Scale (AU)	8.1 ± 8.4	8.9 ± 7.7
Rectus abdominis muscle thickness (cm)	1.3 ± 0.4 (*n* = 13)	1.0 ± 0.2^∗^ (*n* = 15)
Trunk flexor MVC (Nm)	56.4 ± 23.2	50.4 ± 22.5
Trunk flexor time to task failure (s)	755.3 ± 458.4	647.2 ± 339.5
Self-reported physical activity over the previous 12 months (MET^∗^hr^∗^week^-1^)	59.2 ± 38.6 (*n* = 15)	43.2 ± 27.2 (*n* = 17)
Average minutes/day of moderate physical activity	42.0 ± 28.5 (*n* = 5)	37.8 ± 22.7 (*n* = 14)

*^∗^Indicates p < 0.05. cm, centimeters; kg, kilograms; m, meter; Nm, Newton meters; s, seconds; MET, Metabolic equivalents; hr, hour; AU, arbitrary units.*

#### Baseline PPTs Prior to Fatiguing Trunk Flexor Exercise

Men demonstrated higher PPTs than women at the nailbed prior to performance of fatiguing exercise (317.8 ± 162.2 kPa vs. 228.2 ± 90.1 kPa, respectively, *p* = 0.047). Pre-exercise PPTs at the superior rectus abdominis site were similar between men and women (217.7 ± 110.4 kPa vs. 181.5 ± 90.3 kPa, respectively, *p* = 0.294).

#### PPTs Before and After Fatiguing Trunk Flexor Exercise

**FIGURE 2:**
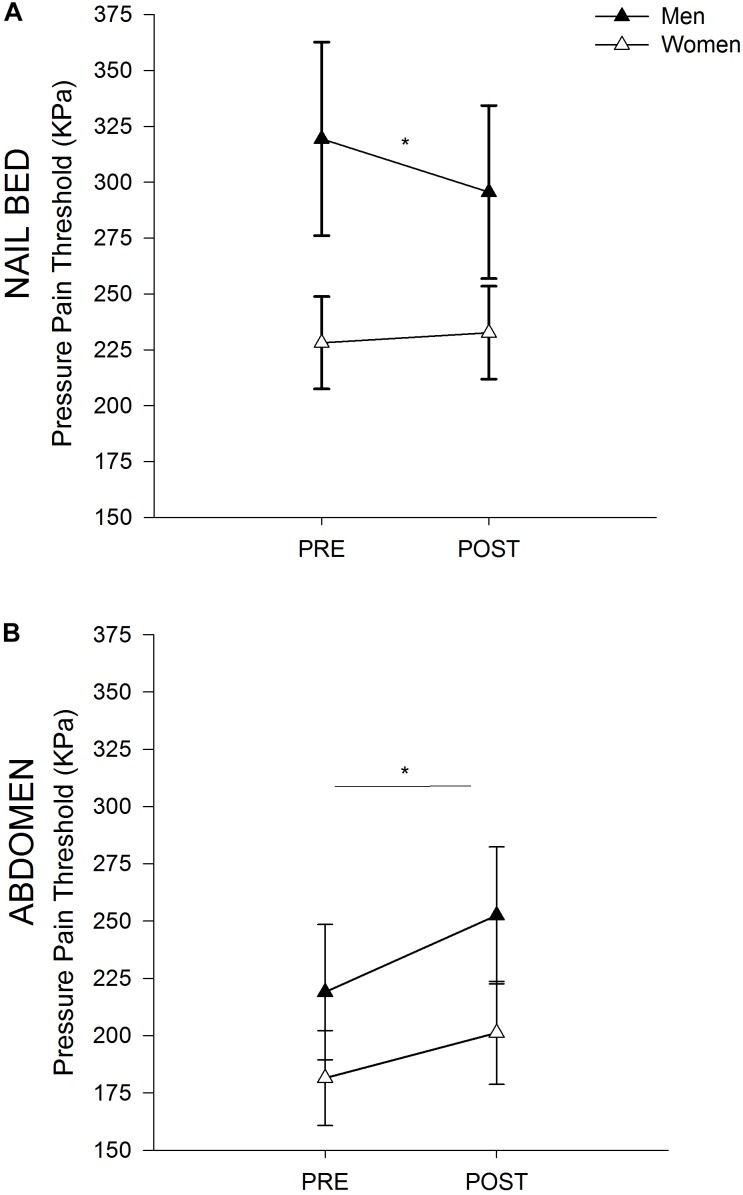
Sex Differences in PPTs. Pressure Pain Threshold before and after Exercise in Men and Women at the Nailbed **(A)** and Abdomen **(B)**. **(A)** Women did not demonstrate a change in PPT at the nailbed following fatiguing trunk flexor exercise, while men demonstrated a decrease in PPT after exercise (hyperalgesia). Men had higher PPTs than women at the nailbed prior to exercise. **(B)** Both men and women demonstrated EIH at the superior rectus abdominis site following fatiguing trunk flexor exercise. Men and women had similar PPTs at the abdomen both before and after exercise. ^∗^ indicates *p* < 0.05 (time effect).

At the rectus abdominis, men and women demonstrated a similar increase in PPTs (i.e., EIH) after fatiguing exercise (Figure 2B; time effect *p* = 0.002; time × sex *p* = 0.575; sex *p* = 0.248). At the nailbed, women demonstrated no change in PPTs following fatiguing exercise, while men demonstrated a small decrease in PPTs (hyperalgesia) (Figure 2A; time effect *p* = 0.054; time × sex *p* = 0.010).

#### Associations

EIH at the abdominal muscle site was positively correlated with rectus abdominis muscle thickness (*r_s_* = 0.462, *p* = 0.013) such that thicker muscle was associated with greater EIH. Baseline PPTs at the nailbed were also associated with the change in PPT after exercise (*r* = -0.443, *p* = 0.008) such that those who had the greatest absolute change in PPT had lower baseline PPT. Furthermore, baseline PPTs at the abdomen were not associated with EIH at the abdomen (*r* = -0.028, *p* = 0.872).

## Discussion

The main and novel findings of this study are: (1) mode of delivery did not affect pain perception or the impact of exercise on pain perception; (2) postpartum women are more sensitive to pain than nulligravid women, especially at the abdomen; (3) EIH was experienced at the abdomen for all groups following fatiguing intermittent isometric exercise of the trunk flexor muscles; (4) men experienced slight hyperalgesia (decrease in PPTs) at the nailbed following trunk flexor fatiguing exercise; and (5) men and women with thicker abdominal muscles reported greater local EIH.

At the initial time point (8–10 weeks postpartum) in protocol 1, postpartum women had lower PPTs at all body sites tested (nailbed, lower abdomen, upper abdomen) than nulligravid women. In contrast, at 26 weeks postpartum, there was no statistical difference in PPTs at the nailbed between postpartum and nulligravid women. This lack of difference at 26 weeks postpartum was likely due to a decline in PPTs in the nulligravid group between the two testing time points because postpartum women demonstrated no change in PPTs between 8 weeks and 26 weeks postpartum. The heightened sensitivity to pain in the postpartum women, which persists at the abdomen almost 6 months after childbirth, highlights the need for assessment and management of pain throughout pregnancy and the postpartum period to avoid developing chronic pain syndromes (Albert et al., 2001; Norén et al., 2002; Phillips and Clauw, 2011). The increased pain sensitivity at the nailbed, and the lack of change in PPT at the nailbed at 26 weeks postpartum suggests that central mechanisms may still be at play in the postpartum group (Staud, 2012). The prevalence of low back and pelvic girdle pain during pregnancy, which often receives minimal treatment, can contribute to central sensitization and the development of chronic pain. Further trauma experienced during labor and delivery (such as surgical intervention, perineal or pelvic floor muscle injury, pelvic joint trauma, etc) and the inflammatory processes which naturally occur as part of the healing process can also contribute to increased sensitization of nociceptors (Chimenti et al., 2018). Further research is needed to understand the role of central sensitization in postpartum women.

To our knowledge, this is the first study to show EIH following fatiguing trunk flexor exercises in any population. The decrease in pain sensitivity (increased PPTs at the abdominal site) following fatiguing trunk flexor exercise experienced by all groups in this study supports the use of abdominal muscle exercise for localized pain relief. This has important clinical considerations because of the prevalence of LBP as well as abdominal pain and incision site pain in postpartum women (Murray and Holdcroft, 1989; Waseem et al., 2011). Furthermore, postpartum women have demonstrated lower trunk flexor strength and lower endurance of both the trunk flexor and lumbopelvic stabilizing muscles compared with nulligravid women (Deering et al., 2018a,b). Thus, incorporating fatiguing exercise of the trunk flexor muscles is especially useful in this population to improve strength and fatigability of this muscle group and to manage pain.

Hypoalgesia was localized to the exercising muscle, which is similar to other studies that report greater EIH at the exercising muscle compared with distal sites (Kosek and Lundberg, 2003). Despite the local EIH effect that was similar between men and women, men reported systemic hyperalgesia (decrease in PPTs) at the nailbed following the trunk flexor exercise while women reported no change in PPTs. Interestingly, sex differences were also present in the baseline pain threshold at the nailbed with men reporting higher PPTs compared with women, and in both protocols baseline pain at the nailbed was associated with the change in PPTs at the nailbed following exercise. We have previously demonstrated similar associations between baseline pain and EIH (Hoeger Bement et al., 2011; Lemley et al., 2016). For example, in women with fibromyalgia, those with lower pain sensitivity (higher PPTs) measured at the finger reported hyperalgesia following exercise (Hoeger Bement et al., 2011). Thus, baseline pain is an important factor in the pain response following exercise; although there may be a critical threshold for this association to occur and may be site specific. For example, the association occurred when baseline pain was measured at the finger or nailbed but not at the abdomen. Furthermore, there was no relation between baseline pain and EIH when baseline PPTs were relatively low (postpartum and nulligravid women) compared to the significant association when men reported higher baseline PPTs than nulligravid women.

Despite men reporting higher PPTs than women at the nailbed, the lack of a sex difference in PPTs at the abdominal site in this study is unique; men typically report higher PPTs compared with women as reviewed by Racine et al. (2012). The lack of a sex difference may be partially explained by the fact that women tend to have greater abdominal fat than men (Deering et al., 2017). Price et al. (2013) showed that higher pain thresholds occur in areas with excess subcutaneous fat.

Another novel finding of this study was that individuals with thicker abdominal muscles demonstrated greater EIH. Previously we have shown that regional lean mass predicts conditioned pain modulation (CPM) (Stolzman and Hoeger Bement, 2016); adolescents with greater lean mass have more efficient descending pain inhibition. We have also shown that CPM is positively associated with EIH (Lemley et al., 2015; Stolzman and Bement, 2016), and the two conditions likely have shared manifestations (Alsouhibani et al., 2018). Therefore, this study provides additional support regarding the importance of lean mass in producing pain relief following exercise potentially via descending pain inhibition.

In summary, fatiguing exercise of the abdominal muscles, using an intermittent isometric protocol, produced localized EIH in healthy postpartum women, nulligravid women, and men. This has important clinical implications because postpartum women demonstrated greater sensitivity to pain than nulligravid women at multiple body sites suggesting the development of central sensitization. There were also sex differences in pain perception at rest and following exercise. Prior to exercise, men reported less pain at the nailbed than women, and only men reported exercise-induced hyperalgesia at this site (distal from the exercising muscle). In both protocols, baseline pain sensitivity at the nailbed was associated with EIH. Thus, this study provides much needed clinical evidence showing that trunk flex exercises produce localized pain relief and the potential role of baseline pain in this response.

## Data Availability

Raw data supporting the conclusions of this manuscript will be made available by the authors, without undue reservation, to any qualified researcher.

## Author Contributions

RD participated in study design, protocol development, procurement of funding, subject recruitment, data collection and analysis, and manuscript writing. TP participated in data collection and analysis and reviewed the manuscript prior to submission. MC participated in procurement of funding, subject recruitment, and manuscript review. SKH participated in study design, protocol development, procurement of funding, data analysis, and manuscript writing. MH participated in study design, protocol development, data analysis, and manuscript writing.

## Conflict of Interest Statement

The authors declare that the research was conducted in the absence of any commercial or financial relationships that could be construed as a potential conflict of interest.
